# Epidemiology of Chikungunya Hospitalizations, Brazil, 2014–2024

**DOI:** 10.3201/eid3109.250554

**Published:** 2025-09

**Authors:** Vaneide Daciane Pedí, Denise Lopes Porto, Wagner de Jesus Martins, Giovanny Vinícius Araújo de França

**Affiliations:** Programa de Pós-Graduação em Medicina Tropical da Faculdade de Medicina da Universidade de Brasília, Brasília, Brazil (V.D. Pedí); Fundação Oswaldo Cruz, Brasília (V.D. Pedí, W.J. Martins); Ministério da Saúde, Brasília (D.L. Porto, G.V.A. de França)

**Keywords:** Chikungunya virus, viruses, vector-borne infections, zoonoses, chikungunya fever, hospitalization, Brazil

## Abstract

We describe 7,421 chikungunya hospitalizations in Brazil covered by the country’s unified health system during 2014–2024. Most (43.2%) hospitalizations occurred in 2016 and 2017, reaching 0.72 (95% CI 0.69–0.76) hospitalizations/100,000 population in 2016. Hospitalizations were more frequent among persons who were female (55.8%), identifying as brown or black (63.5%), and 1–19 years of age (31.4%). Intensive care unit admissions occurred in 1.4% of cases, predominantly among children <5 and adults >85 years of age. The overall in-hospital case-fatality rate was 1.1%, which increased substantially with age, reaching 11.5% among patients >90 years of age and 14.1% among men 85–89 years of age. Patients admitted to the intensive care unit had a case-fatality rate of 21.1%. The total cost of chikungunya hospitalizations was US $560,746 (US $76.26 per patient). Our findings provide insights for surveillance of the most severe chikungunya cases.

Chikungunya virus (CHIKV), an alphavirus of the Togaviridae family, is transmitted to humans by *Aedes* spp. mosquitoes, primarily *Ae. aegypti* and *Ae. albopictus* mosquitoes, and causes chikungunya in humans, commonly in tropical and subtropical regions worldwide (*1*). Phylogenetic analyses have categorized CHIKV into 3 main lineages: the East/Central/South African (ECSA) lineage, the West African lineage, and the Asian lineage (*2*,*3*). 

By December 2022, CHIKV had been detected in >110 countries in Asia, Africa, Europe, and the Americas, and all regions with established *Ae. aegypti* or *Ae. albopictus* mosquito populations have reported local transmission of the virus (*4*). Since 2016, Brazil has been the epicenter of chikungunya epidemics in the Americas, and 1,700,762 suspected cases were reported nationwide during 2017–2024 (*5*). The country has a sizeable susceptible population, a favorable climate, and abundant populations of *Ae. aegypti* mosquitoes, which could contribute to the occurrence of rapid and localized chikungunya outbreaks, and high CHIKV infection rates are followed by periods of lower chikungunya incidence (*5*). In addition, seropositivity is highly heterogeneous in Brazil, and estimates range from 7.4% to 51% (*6*,*7*).

Chikungunya fever refers to the acute illness caused by CHIKV. Most (75%–95%) infected persons develop symptoms, which usually begin within 4 to 8 days after the bite of a CHIKV-infected mosquito. Symptoms can include fever, myalgia, and arthralgia, and CHIKV infection can cause decompensation of underlying conditions (*8*). Although chikungunya illness is generally self-limited, some patients—especially infants; older adults; and persons with underlying conditions, co-infections, or certain genetic traits—can develop atypical or severe forms. Those forms can involve neurologic disturbances, cardiovascular complications, hemorrhagic signs, or Guillain-Barré syndrome (*9*–*11*). The reported frequency of such manifestations varies across outbreaks and populations, from 0.3% during the 2005–2006 Réunion Island epidemic to 9.8% among hospitalized patients in French Guiana during 2014–2015 (*12*,*13*).

In 30%–40% of patients, chikungunya becomes chronic, persisting for >3 months and causing arthritis, fatigue, sleep disorders, myalgia, skin lesions, depression, and digestive disorders (*1*,*14*,*15*). Among the severe manifestations of CHIKV infection, cases of meningoencephalitis, bullous skin lesions, multiple organ failure with hemorrhage, and sepsis have been reported (*16*,*17*). Furthermore, CHIKV infection increasingly has been associated with substantial mortality rates, particularly because of complications, such as heart or cerebrovascular disease, renal impairment, cardiogenic or septic shock, or decompensated diabetes (*18*–*20*).

Data from outbreaks in different countries show that 0.6%–13.0% of patients with confirmed chikungunya are hospitalized (*1*,*8*,*10*,*21*). In Brazil, the unified health system, Sistema Único de Saúde (SUS), provides universal access to healthcare, and in 2019, SUS covered almost 65% of all hospitalizations in the country (*22*). The SUS hospital information system, Sistema de Informação Hospitalar/SUS (SIH/SUS), is the country’s only source of information on hospitalizations. The system is mainly used to define the values for payment of medium- and high-complexity services provided by healthcare establishments throughout the country (*23*). We used SIH/SUS data to explore the epidemiology of chikungunya hospitalizations in Brazil during 2014­–2024, focusing on patient demographic characteristics, spatiotemporal dynamics, and costs covered by SUS.

## Materials and Methods

**Figure 1:**
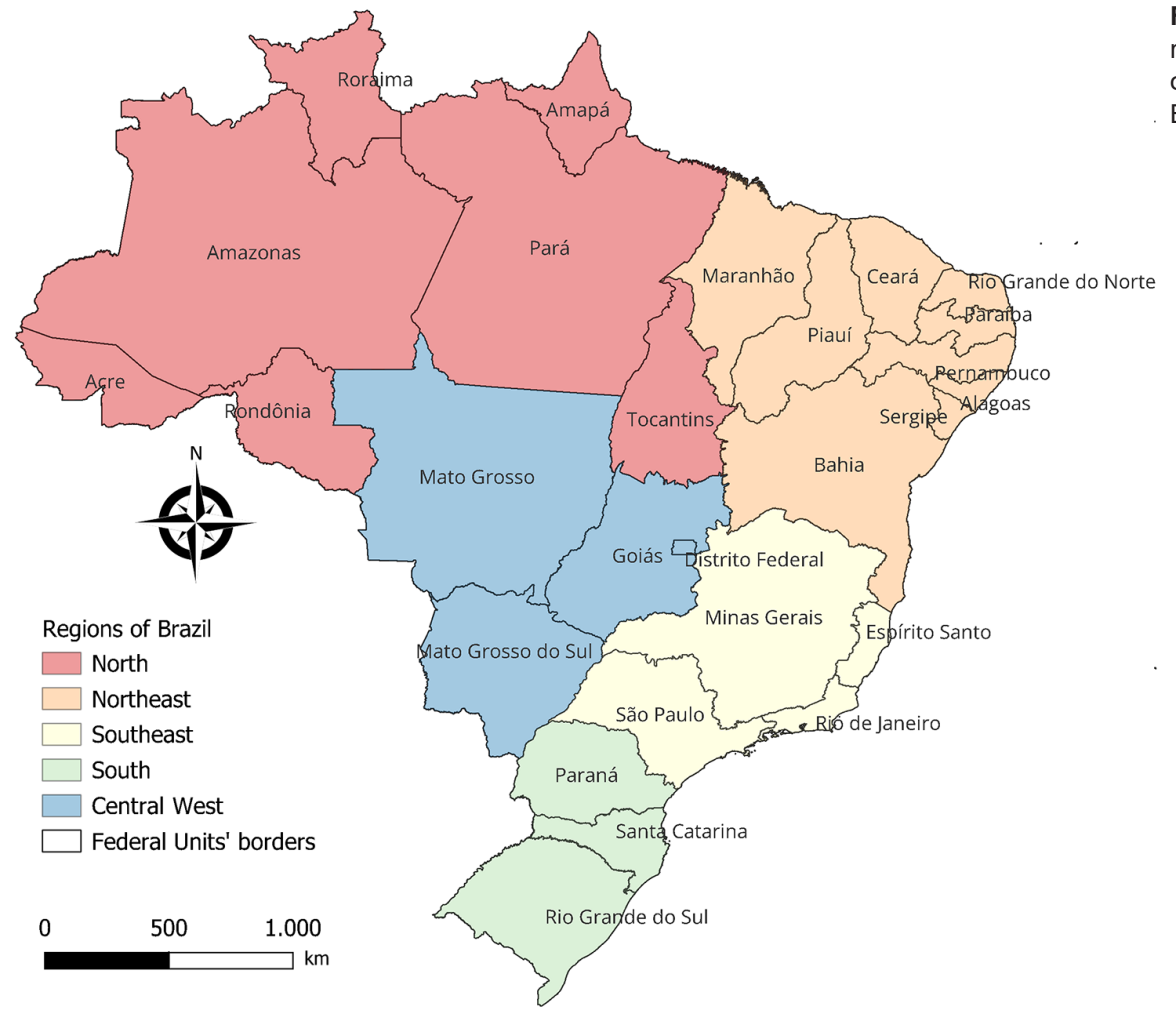
Regions and federal units referenced in study of epidemiology of chikungunya hospitalizations, Brazil, 2014–2024.

We conducted a cross-sectional descriptive study of chikungunya hospitalizations in Brazil, its 5 regions, and 26 states and the Federal District (Figure 1), using publicly available SIH/SUS data from Datasus (https://datasus.saude.gov.br/transferencia-de-arquivos). The SIH/SUS data reflect only hospitalizations covered by the public healthcare system and do not capture costs from private sector hospitalizations or out-of-pocket expenses.

We used data on chikungunya hospitalizations financed by SUS and registered in the SIH/SUS during January 1, 2014–December 31, 2024. We selected all hospitalizations that had chikungunya as the primary diagnosis and were registered with code A92.0 from the International Classification of Diseases, 10th Revision (ICD-10), as adopted by the Ministry of Health (*24*). Secondary diagnoses (i.e., conditions that coexisted at the time of admission) were only available in SIH/SUS dataset for 2014, and chikungunya was not reported as a secondary diagnosis during 2015–2024; therefore, our analysis was limited to chikungunya as the primary diagnosis.

We used the following variables in our analyses: year and month of hospitalization; sex; age in years, estimated from the patient’s date of birth and the date of hospitalization, classified into 5-year age groups; race/skin color, according to patients’ self-declarations and classified into white, black, brown, Asian, and Indigenous (*25*); and intensive care unit (ICU) admission and death, when applicable. We calculated length of hospital stay in daily rates, considering the patient’s stay in a hospital institution for an indivisible period of <24 hours and assuming midnight as the reference time for the start and end of the period for the daily rate, as defined by SIH/SUS.

We estimated case-fatality rates (CFRs) by dividing the number of deaths resulting from chikungunya by the number of chikungunya hospitalizations for each year. To calculate crude hospitalization rates, we used the annual population estimates based on the 2022 demographic census for Brazil (*26*). Thus, we defined crude hospitalization rates by region and state as the ratio between the number of chikungunya hospitalizations and the total population in the year, multiplied by 100,000 population. We calculated crude chikungunya hospitalization rates by year, region, state, sex, and age group.

We standardized annual hospitalization rates by age through the direct method, using the World Health Organization global standard population (*27*). We initially calculated the annual age-adjusted rate by multiplying each age-specific hospitalization rate times the world standard population in the corresponding age group. We summed the products across all age groups and divided by the total standard population to estimate the overall annual age-standardized hospitalization rate. We used R version 4.4.1 (The R Project for Statistical Computing, https://www.r-project.org) and the dsr package (https://cran.r-project.org/src/contrib/Archive/dsr) to calculate standardized hospitalization rates and 95% CIs by applying a method based on gamma distribution.

To describe the chikungunya-related hospitalization costs covered by the government of Brazil through SUS that were recorded in the SIH/SUS database, we considered the total hospitalization cost and its 2 components: professional services, which comprise doctors’ and dentists’ fees; and hospital services, which include daily rates, room rates, food, and other services. We also assessed costs of daily rates for patients admitted to the ICU. 

We described quantitative data by using absolute and relative frequencies and quantitative variables through measures of central tendency (mean and median) and dispersion (SD and interquartile range [IQR]). We converted cost estimates from Brazilian real (BRL) to US dollars (USD) by using annual exchange rates on July 1 of each year. We also provide the annual cost estimates in BRL adjusted for inflation to July 1, 2024, values by using the Consumer Price Index for Brazil, and we converted to USD by using the exchange rate on July 1, 2014.

We analyzed chikungunya hospitalizations registered in SIH/SUS compared with the total number of suspected and confirmed chikungunya cases notified in the Notifiable Diseases Information System (SINAN), applying the case definition adopted by the Ministry of Health (*28*) and using aggregated data available in Tabnet (*5*). Although publicly available data are restricted to chikungunya cases notified from 2017 onward, the SINAN system allows retroactive case registration and records both notification and symptom onset dates. Thus, we included all cases notified from 2017 onward that had symptom onset from January 2014 onward.

We performed data analysis by using TabWin version 4.1.5 (http://www.portalsinan.saude.gov.br/sistemas-auxiliares/tabwin), Stata version 13.0 (StataCorp LLC, https://www.stata.com), R version 4.4.1, and Microsoft Office 2024 (https://www.microsoft.com). Because we used anonymized and publicly available data, we were not required to submit the project for evaluation by a research ethics committee, as established by Resolution CNS/MS no. 510/2016.

## Results

During 2014–2024, Brazil recorded 7,421 chikungunya hospitalizations in SIH/SUS, which corresponded to 0.4% of 1,698,976 suspected cases and 0.9% of 830,386 cases confirmed by laboratory or clinical-epidemiologic findings that were recorded in SINAN during the same timeframe and considering date of symptom onset. Hospitalizations followed the pattern of suspected and confirmed case curves during 2017–2024 (Appendix Figure 1).

**Figure 2:**
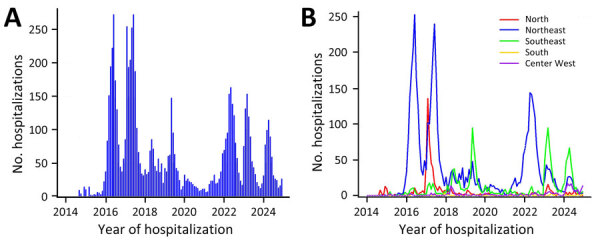
Number of hospitalizations per year in study of epidemiology of chikungunya hospitalizations, Brazil, 2014–2024. A) Total number of hospitalizations per year; B) number of hospitalizations per year by region.

Most (43.2%) hospitalizations occurred in 2016 and 2017, and we noted peaks in June 2016 (272 hospitalizations), February 2017 (254 hospitalizations), June 2017 (272 hospitalizations), May 2019 (147 hospitalizations), May 2022 (163 hospitalizations), March 2023 (153 hospitalizations), and April 2024 (114 hospitalizations) (Figure 2, panel A). Cases in the Northeast region accounted for peaks in June 2016 (253/272 cases) and June 2017 (240/272), comprising 93.0% of hospitalizations in June 2016 and 88.2% in June 2017. In addition, 88.3% of hospitalizations in May 2022 occurred in the Northeast region. In February 2017, the North region accounted for 53.5% of hospitalizations. In the Southeast region, peaks occurred in May 2019 (95 cases), March 2023 (95 cases), and April 2024 (67 cases) (Figure 2, panel B).

In Brazil, the highest crude hospitalization rates were in 2017 at 0.84 (95% CI 0.80–0.88)/100,000 population, and 2016 at 0.72 (95% CI 0.69–0.76)/100,000 population; we observed a smaller peak of 0.49 (95% CI 0.46–0.52)/100,000 population in 2022. Standardized rates closely mirrored crude rates, with minor decimal differences; therefore, we decided to focus on the crude hospitalization rates (Table 1).

**Table 1:** Annual crude and age-standardized hospitalization rates from a study of epidemiology of chikungunya hospitalizations, Brazil, 2014–2024

Year	No. hospitalizations	Population	Crude hospitalization rate (95% CI)	Age-standardized hospitalization rate (95% CI)
2014	27	200,811,131	0.013 (0.009–0.020)	0.013 (0.009–0.019)
2015	76	202,403,642	0.038 (0.030–0.047)	0.039 (0.030–0.049)
2016	1,476	203,871,925	0.724 (0.688–0.762)	0.730 (0.693–0.769)
2017	1,729	205,211,557	0.843 (0.803–0.883)	0.868 (0.827–0.910)
2018	577	206,529,038	0.279 (0.257–0.303)	0.279 (0.257–0.303)
2019	635	207,900,099	0.305 (0.282–0.330)	0.307 (0.284–0.332)
2020	211	209,164,889	0.101 (0.088–0.115)	0.101 (0.088–0.116)
2021	286	210,103,642	0.136 (0.121–0.153)	0.142 (0.126–0.160)
2022	1,037	210,862,983	0.492 (0.462–0.523)	0.482 (0.452–0.513)
2023	762	211,695,158	0.360 (0.335–0.386)	0.351 (0.326–0.377)
2024	605	212,583,750	0.285 (0.262–0.308)	0.281 (0.258–0.305)

**Figure 3:**
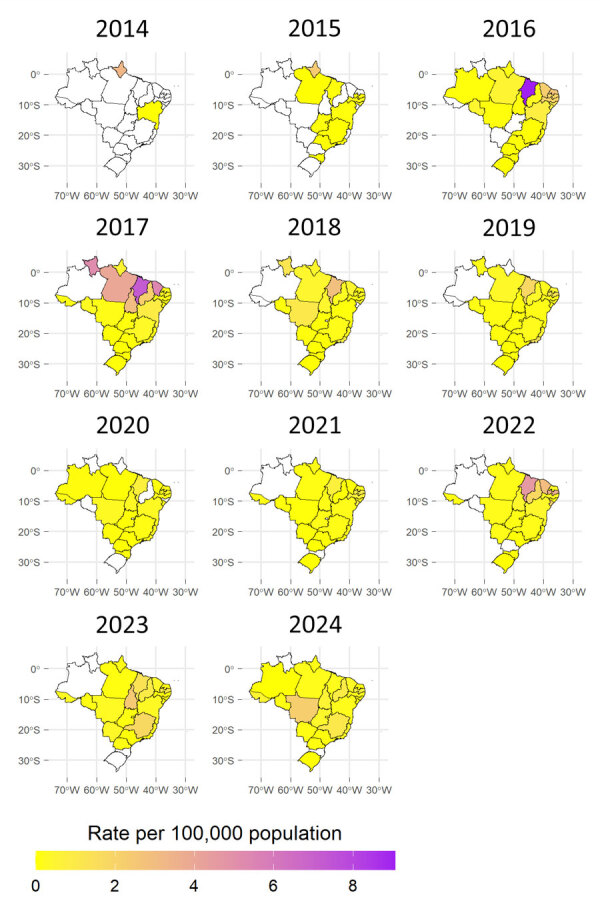
Annual hospitalization rates per region from study of epidemiology of chikungunya hospitalizations, Brazil, 2014–2024.

Among regions, the Northeast had the highest hospitalization rate in 6 of the 11 years analyzed, reaching the highest rate in 2016 (2.46 hospitalizations/100,000 population), and the states of Maranhão (9.05/100,000 population) and Rio Grande do Norte (2.66/100,000 population) had the highest rates that year (Table 2). Maranhão had the highest hospitalization rates in 2018 (2.86/100,000 population) and 2022 (4.6/100,000 population) (Figure 3; Appendix Figure 2).

**Table 2:** Crude hospitalization rates by region and state in a study of epidemiology of chikungunya hospitalizations, Brazil, 2014–2024*

Region, state	No. hospitalizations (rate/100,000 population)										
	2014	2015	2016	2017	2018	2019	2020	2021	2022	2023	2024
North											
Acre	0	0	0	1 (0.12)	0	0	1 (0.12)	1 (0.12)	1 (0.11)	2 (0.23)	4 (0.45)
Amapá	24 (3.26)	18 (2.41)	1 (0.13)	4 (0.52)	3 (0.39)	0	0	1 (0.13)	1 (0.13)	1 (0.13)	1 (0.12)
Amazonas	0	0	2 (0.05)	0	0	0	3 (0.07)	0	0	0	1 (0.02)
Pará	0	1 (0.01)	38 (0.46)	330 (3.99)	35 (0.42)	26 (0.31)	3 (0.04)	9 (0.11)	13 (0.15)	7 (0.08)	15 (0.17)
Rondônia	0	0	7 (0.42)	4 (0.42)	1 (0.06)	2 (0.12)	0	1 (0.06)	0	2 (0.11)	2 (0.11)
Roraima	0	0	0	29 (5.14)	7 (1.19)	1 (0.16)	0	0	0	0	0
Tocantins	0	0	1 (0.07)	45 (3)	6 (0.4)	1 (0.07)	4 (0.26)	2 (0.13)	6 (0.39)	38 (2.42)	8 (0.51)
Total	24 (0.14)	19 (0.11)	49 (0.28)	413 (2.35)	52 (0.29)	30 (0.17)	11 (0.06)	14 (0.08)	21 (0.11)	50 (0.27)	31 (0.17)
Northeast											
Alagoas	0	7 (0.22)	43 (1.35)	4 (0.13)	9 (0.28)	6 (0.19)	0	4 (0.12)	3 (0.09)	3 (0.09)	3 (0.09)
Bahia	2 (0.01)	9 (0.06)	112 (0.77)	124 (0.85)	8 (0.05)	17 (0.12)	38 (0.26)	22 (0.15)	57 (0.38)	41 (0.28)	25 (0.17)
Ceará	0	0	201 (2.27)	484 (5.42)	39 (0.43)	16 (0.18)	5 (0.06)	2 (0.02)	273 (2.98)	31 (0.34)	28 (0.3)
Maranhão	0	4 (0.06)	621 (9.05)	497 (7.21)	198 (2.86)	127 (1.83)	47 (0.67)	47 (0.67)	366 (3.2)	93 (1.33)	12 (0.17)
Paraíba	0	1 (0.03)	77 (1.95)	13 (0.33)	12 (0.3)	50 (1.24)	9 (0.22)	43 (1.05)	148 (3.6)	45 (1.09)	32 (0.77)
Pernambuco	0	14 (0.15)	192 (2.07)	8 (0.09)	8 (0.09)	11 (0.12)	14 (0.15)	59 (0.62)	20 (0.21)	4 (0.04)	14 (0.15)
Piauí	0	0	7 (0.21)	71 (2.16)	8 (0.24)	12 (0.36)	0	1 (0.03)	59 (1.76)	31 (0.92)	30 (0.89)
Rio Grande do Norte	0	0	89 (2.66)	5 (0.15)	2 (0.06)	16 (0.47)	5 (0.15)	5 (0.15)	8 (0.23)	5 (0.15)	2 (0.06)
Sergipe	0	0	22 (1)	1 (0.05)	0	2 (0.09)	2 (0.09)	21 (0.93)	54 (2.38)	3 (0.13)	1 (0.04)
Total	2 (0.01)	35 (0.06)	1,364 (2.46)	1,207 (2.17)	284 (0.51)	257 (0.46)	120 (0.21)	204 (0.36)	944 (1.66)	256 (0.45)	147 (0.26)
Southeast											
Espírito Santo	0	0	2 (0.05)	3 (0.08)	6 (0.15)	15 (0.38)	9 (0.22)	7 (0.17)	2 (0.05)	10 (0.25)	17 (0.41)
Minas Gerais	0	2 (0.01)	7 (0.03)	59 (0.29)	30 (0.14)	37 (0.18)	20 (0.1)	28 (0.13)	32 (0.15)	376 (1.77)	223 (1.05)
Rio de Janeiro	0	4 (0.02)	27 (0.16)	15 (0.09)	147 (0.86)	256 (1.49)	22 (0.13)	4 (0.02)	6 (0.03)	9 (0.05)	31 (0.18)
São Paulo	1 (0)	10 (0.02)	16 (0.04)	14 (0.03)	13 (0.03)	21 (0.05)	12 (0.03)	13 (0.03)	11 (0.02)	15 (0.03)	29 (0.06)
Total	1 (0)	16 (0.02)	52 (0.06)	91 (0.11)	196 (0.23)	329 (0.38)	63 (0.07)	52 (0.06)	51 (0.06)	410 (0.46)	300 (0.34)
South											
Paraná	0	0	2 (0.02)	2 (0.02)	2 (0.02)	1 (0.01)	1 (0.01)	2 (0.02)	0	12 (0.1)	9 (0.08)
Rio Grande do Sul	0	0	0	5 (0.04)	1 (0.01)	2 (0.02)	0	2 (0.02)	1 (0.01)	0	1 (0.01)
Santa Catarina	0	1 (0.01)	2 (0.03)	1 (0.01)	1 (0.01)	5 (0.07)	3 (0.04)	0	2 (0.03)	0	0
Total	0	1 (0)	4 (0.01)	8 (0.03)	4 (0.01)	8 (0.03)	4 (0.01)	4 (0.01)	3 (0.01)	12 (0.04)	10 (0.03)
Center West											
Distrito Federal	0	2 (0.07)	5 (0.18)	1 (0.04)	0	1 (0.03)	0	0	1 (0.03)	10 (0.34)	2 (0.07)
Goiás	0	3 (0.05)	0	4 (0.06)	3 (0.04)	4 (0.06)	2 (0.03)	9 (0.13)	7 (0.1)	6 (0.08)	27 (0.37)
Mato Grosso	0	0	2 (0.06)	4 (0.14)	36 (1.03)	4 (0.11)	9 (0.25)	2 (0.05)	6 (0.16)	9 (0.24)	85 (2.22)
Mato Grosso do Sul	0	0	0	1 (0.04)	2 (0.07)	2 (0.07)	2 (0.07)	1 (0.04)	4 (0.14)	9 (0.31)	3 (0.21)
Total	0	5 (0.03)	7 (0.04)	10 (0.06)	41 (0.26)	11 (0.07)	13 (0.08)	12 (0.07)	18 (0.11)	34 (0.2)	117 (0.69)
Brazil total	27 (0.01)	76 (0.04)	1,476 (0.72)	1,729 (0.84)	577 (0.28)	635 (0.31)	211 (0.1)	286 (0.14)	1,037 (0.49)	762 (0.36)	605 (0.28)


*Information obtained from the Brazil Ministry of Health Datasus (https://datasus.saude.gov.br/transferencia-de-arquivos).

Overall, most (55.8%) hospitalized chikungunya patients were female, and 63.5% of chikungunya hospitalizations were among persons who identified as brown or black, a pattern observed throughout the evaluated years. The median age of hospitalized patients was 34 (IQR 12–58) years and ranged from 27 years in 2015 (IQR 6–48 years) and 2021 (IQR 9–49 years) to 43 (IQR 13–65) years in 2024. Cases were concentrated among persons 1–19 years of age, who comprised 31.4% of overall hospitalizations. In 2017, the year with the highest number of hospitalizations, the predominant age groups were 1–9 years (236 [23%] hospitalizations), 10–19 years (205 [13.9%]), and 30–39 years (157 [10.6%]) (Table 3; Appendix Tables 1, 2).

**Table 3:** Patient characteristics from 7,421 chikungunya hospitalizations covered by the unified health system, Brazil, 2014–2024*

Characteristics	Year, no. (%)											
	Total	2014	2015	2016	2017	2018	2019	2020	2021	2022	2023	2024
Sex												
M	3,282 (44.2)	10 (37)	34 (44.7)	620 (42)	787 (45.5)	266 (46.1)	285 (44.9)	104 (49.3)	130 (45.5)	441 (42.5)	340 (44.6)	265 (43.8)
F	4,139 (55.8)	17 (63)	42 (55.3)	856 (58)	942 (54.5)	311 (53.9)	350 (55.1)	107 (50.7)	156 (54.5)	596 (57.5)	422 (55.4)	340 (56.2)
Race, ethnicity												
White	1,009 (13.6)	2 (7.4)	18 (23.7)	160 (10.8)	140 (8.1)	90 (15.6)	164 (25.8)	30 (14.2)	37 (12.9)	77 (7.4)	139 (18.2)	152 (25.1)
Black	182 (2.5)	0	3 (3.9)	19 (1.3)	33 (1.9)	20 (3.5)	32 (5)	8 (3.8)	2 (0.7)	6 (0.6)	35 (4.6)	24 (4.0)
Brown	4,526 (61)	5 (18.5)	20 (26.3)	815 (55.2)	1,099 (63.6)	337 (58.4)	293 (46.1)	133 (63)	153 (53.5)	742 (71.6)	533 (69.9)	396 (65.5)
Asian	360 (4.9)	0	1 (1.3)	148 (10)	104 (6)	29 (5)	34 (5.4)	3 (1.4)	2 (0.7)	2 (0.2)	9 (1.2)	28 (4.6)
Indigenous	9 (0.1)	0	0	1 (0.1)	0	2 (0.3)	1 (0.2)	0	0	0	0	5 (0.8)
Not reported	1,335 (18)	20 (74.1)	34 (44.7)	333 (22.6)	353 (20.4)	99 (17.2)	111 (17.5)	37 (17.5)	92 (32.2)	210 (20.3)	46 (6)	0
Age group, y												
<1	228 (3.1)	2 (7.4)	10 (13.2)	56 (3.8)	59 (3.4)	10 (1.7)	17 (2.7)	3 (1.4)	22 (7.7)	26 (2.5)	8 (1)	15 (2.5)
1–9	1,262 (17.0)	1 (3.7)	15 (19.7)	236 (16)	386 (22.3)	71 (12.3)	89 (14)	35 (16.6)	56 (19.6)	147 (14.2)	119 (15.6)	107 (17.7)
10–19	1,072 (14.4)	4 (14.8)	10 (13.2)	205 (13.9)	297 (17.2)	88 (15.3)	110 (17.3)	23 (10.9)	46 (16.1)	120 (11.6)	96 (12.6)	73 (12.1)
20–29	796 (10.7)	6 (22.2)	5 (6.6)	154 (10.4)	154 (8.9)	93 (16.1)	73 (11.5)	40 (19)	32 (11.2)	115 (11.1)	75 (9.8)	49 (8.1)
30–39	799 (10.8)	6 (22.2)	8 (10.5)	157 (10.6)	183 (10.6)	95 (16.5)	76 (12)	31 (14.7)	33 (11.5)	99 (9.5)	71 (9.3)	40 (6.6)
40–49	770 (10.4)	3 (11.1)	10 (13.2)	151 (10.2)	157 (9.1)	90 (15.6)	71 (11.2)	21 (10)	26 (9.1)	112 (10.8)	75 (9.8)	54 (8.9)
50–59	734 (9.9)	2 (7.4)	10 (13.2)	126 (8.5)	152 (8.8)	53 (9.2)	76 (12)	16 (7.6)	26 (9.1)	124 (12)	75 (9.8)	74 (12.2)
60–69	657 (8.9)	1 (3.7)	2 (2.6)	125 (8.5)	122 (7.1)	36 (6.2)	64 (10.1)	17 (8.1)	19 (6.6)	117 (11.3)	74 (9.7)	80 (13.2)
70–79	578 (7.8)	1 (3.7)	3 (3.9)	133 (9)	120 (6.9)	22 (3.8)	35 (5.5)	17 (8.1)	18 (6.3)	97 (9.4)	72 (9.4)	60 (9.9)
80–89	412 (5.6)	1 (3.7)	2 (2.6)	105 (7.1)	77 (4.5)	16 (2.8)	21 (3.3)	7 (3.3)	4 (1.4)	64 (6.2)	73 (9.6)	42 (6.9)
>90	113 (1.5)	0	1 (1.3)	28 (1.9)	22 (1.3)	3 (0.5)	3 (0.5)	1 (0.5)	4 (1.4)	16 (1.5)	24 (3.1)	11 (1.8)
ICU admission												
N	7,317 (98.6)	27 (100)	74 (97.4)	1,466 (99.3)	1,715 (99.2)	571 (99)	624 (98.3)	206 (97.6)	281 (98.3)	1,023 (98.6)	746 (97.9)	584 (96.5)
Y	104 (1.4)	0	2 (2.6)	10 (0.7)	14 (0.8)	6 (1)	11 (1.7)	5 (2.4)	5 (1.7)	14 (1.4)	16 (2.1)	21 (3.5)
Death												
N	7,337 (98.9)	27 (100)	75 (98.7)	1,449 (98.2)	1,709 (98.8)	576 (99.8)	631 (99.4)	206 (97.6)	286 (100)	1,028 (99.1)	754 (99)	596 (98.5)
Y	84 (1.1)	0	1 (1.3)	27 (1.8)	20 (1.2)	1 (0.2)	4 (0.6)	5 (2.4)	0	9 (0.9)	8 (1)	9 (1.5)

*Information obtained from the Brazil Ministry of Health Datasus (https://datasus.saude.gov.br/transferencia-de-arquivos). ICU, intensive care unit.

When we stratified annual crude hospitalization rates by sex and age group, we observed similar patterns for male and female sex in age groups from 0 to 79 years, with few exceptions (Appendix Figures 3, 4). However, we observed notable differences in the 80–84, 85–89, and >90 age groups. For 2016, 2018, 2022, and 2023, we observed higher hospitalization rates among men 80–84 years of age compared with women in the same age group, and the largest difference was in 2019, when the hospitalization rate in men was 4 times higher than that observed among women (1.29 vs. 0.29/100,000 population) (Appendix Figures 3, 4). For persons 85–89 years of age, we observed the highest hospitalization rate in 2016, when the rate for men was nearly twice that of women (4.25 vs. 2.5/100,000 population). Persons >90 years of age had the highest recorded hospitalization rates during the study period, reaching 9.22/100,000 population among men in 2016, nearly 4 times higher than among women (2.66/100,000 population) (Appendix Figure 4).

Overall, the average length of hospital stay for chikungunya patients was 3.8 + 0.1 days, and the median stay was 3 (IQR 2–4) days, without substantial variations by sex or age during the study period (Appendix Table 1). Of note, patients admitted to the ICU had longer stays of 13.1 + 1.1 days than those not admitted to the ICU (3.6 + 0.1 days). In addition, patients who died during hospitalization also had longer average stays (8.0 + 1.5 days) than patients who survived (3.7 + 0.1 days).

Among all 7,421 chikungunya hospitalizations, 104 (1.4%) patients were admitted to the ICU, and that population was equally divided by male and female patients, 52 (50.0%) in each group. The age groups with the highest ICU admissions were adults >85 years of age (3.3%) and children <5 years of age (2.7%). The ages of patients admitted to the ICU varied greatly; the mean was 38.6 + 31.3 years, and the median was 37.5 (IQR 7.5–69) years. The highest percentages of ICU admissions were recorded in 2024 (3.5%) and 2023 (2.1%); admissions were more frequent in children <5 years of age (2.7%), but rates were similar among boys (2.7%) and girls (2.6%). Among persons >60 years of age, 1.9% were admitted to the ICU, predominantly men (2.3% vs. 1.6% women) (Table 3; Appendix Table 2).

Among the 84 hospitalized patients who died, 54.8% were male, 45.2% were female, and 26.4% were >80 years of age (mean 69.4 + 25.8 years; median 79.0, IQR 62–86 years). The highest CFRs were recorded in 2020 (2.4%) and 2016 (1.8%) and substantially increased among persons >60 years of age, reaching 10.3% among persons 85–89 years of age and 11.5% among persons >90 years of age. Estimated rates were higher among men than women for the 85–89 age group (14.1% vs. 6.41%) and the >90 age group (13.7% vs. 9.7%). In addition, the CFRs was higher among persons admitted to the ICU than those who were not (21.1% vs. 0.8%) (Table 3; Appendix Table 3).

During 2014–2024, the total cost of chikungunya hospitalizations covered by SUS was ≈US $560,746 and the average cost per patient was ≈US $76.26 (median $47.38) (Appendix Tables 4, 5). After adjusting for inflation to 2024 values, total hospitalization costs for chikungunya in BRL increased by 26.7%, from BRL $2.28 million to BRL $2.89 million. However, when we used the 2024 exchange rate (1 BRL = US $0.179036) to convert inflation-adjusted values to USD, the total cost decreased to US $516,921.61, reflecting the cumulative depreciation of the BRL relative to the USD over the study period (Appendix Table 6).

The highest total hospitalization costs were recorded in 2017 (US $120,960.10) and 2016 (US $120,336.60). In contrast, the highest average costs per patient were observed in 2024 (US $85.69) and 2020 (US $84.24). Across the study period, hospital services accounted for 84.2% of the total chikungunya-related hospitalization costs, ranging from 79.5% in 2014 to 85.8% in 2020. ICU costs corresponded to 19.6% of the total hospitalization expenses during 2014–2024, reaching 42.1% in 2020 (Appendix Table 4).

## Discussion

This study examined chikungunya-related hospitalizations in Brazil since 2014­, the year when the first autochthonous cases were reported in Oiapoque (Amapá state) and in Feira de Santana (Bahia state) (*29*). SIH/SUS data show ≈0.4% of suspected and ≈0.9% of confirmed chikungunya cases required hospitalization during 2014–2024, consistent with previous estimates of 0.6%–13.0% reported during outbreaks in various countries (*1*,*10*,*21*). Moreover, the pattern of chikungunya-related hospitalizations followed epidemic peaks in Brazil, particularly in the Northeast (2016, 2017, and 2022), North (2017), and Southeast (2019, 2023, and 2024) regions; we also noted an increase in the Central West region in 2024. Throughout the study period, publications documented chikungunya outbreaks in Northeast states, including in Maranhão (*30*), Bahia (*31*), and Alagoas (*32*,*33*), as well as in the North (*34*) and Southeast regions (*35*,*36*). Although cases have been reported in the South, most were likely imported from other regions (*37*). The increase in suspected and confirmed chikungunya cases since 2022 reflects multiple factors, including increased transmission in several states, particularly in the Southeast and Central West regions, and improved diagnostic capacity and clinical awareness. During the COVID-19 pandemic, disruptions in surveillance and healthcare services, combined with the prioritization of hospital resources for COVID-19 patients, likely contributed to underreporting of arboviral diseases and lower chikungunya hospitalization rates during 2020–2021.

We observed higher hospitalization rates among men >80 years of age compared with women in the same age groups, consistent with a study in Martinique and Guadeloupe from 2013–2015, where older adults, particularly persons >75 years of age, had increased hospitalization rates and ICU admissions (*10*). That study also reported an ICU admission rate of 7.4% among hospitalized patients, which is substantially higher than the 1.4% rate observed in Brazil during 2014–2024.

We observed an overall CFR of 1.1%, and CFR peaked at 2.4% in 2020. In another study that used data linked between the SINAN and Brazil’s mortality information system (SIM) for 2016 and 2017 (*38*), estimated CFR was 0.08% (0.8 death/1,000 cases) based solely on SINAN data. After adjusting for underreporting using SIM data, the corrected CFR increased to 0.57% (5.7 deaths/1,000 cases) (*38*). Our higher rate reflects the focus on hospitalized cases, which represent more severe clinical presentations.

In our study, CFR was substantially higher in men >85 years of age, and ICU admission correlated with CFR, which supports the hypothesis that those patients experienced more severe illness. During the 2014–2015 chikungunya outbreak in French Polynesia, 64 patients with confirmed chikungunya infection were admitted to ICUs, of whom 21 (32.8%) had severe sepsis or septic shock develop and 18 (28.1%) died (*39*). A 2025 meta-analysis also identified male sex, age >60 years, and chronic diseases, particularly diabetes mellitus, hypertension, and chronic kidney disease, as risk factors for chikungunya-related death (*20*). In our study, lack of data on underlying conditions in SIH/SUS precluded further risk assessment.

Among our cohort, 62 deaths occurred without ICU admission, which might indicate a lack of available ICU beds in the healthcare network or challenges in case management and referral processes. Furthermore, no deaths were recorded among persons 5–19 years of age, but ICU admissions among children <5 years reached 2.3% and CFR was 0.6% in that age group. Those findings are consistent with a previous study (*40*) that indicated that children, particularly children <6 months of age, are more susceptible to severe chikungunya complications, including neurologic and cardiac involvement, often leading to hospitalization (*40*).

Overall, the actual mortality burden of chikungunya is likely underreported because of limited clinical suspicion and co-circulating arboviruses, such as dengue. The extent of underreporting has been estimated to reach as high as 98% in some settings. For instance, in Minas Gerais, Brazil, the number of excess deaths was estimated to be 60 times higher than confirmed chikungunya deaths in 2023 (*41*); Pernambuco, Brazil, recorded 4,505 excess deaths in 2016 compared with 94 deaths officially attributed to chikungunya (*42*); and Puerto Rico identified 1,310 excess chikungunya deaths in 2014, but only 31 were confirmed through routine surveillance (*43*).

Another study analyzed hospitalizations from all causes in the SIH/SUS database from July 2018–June 2019 (*44*). That study reported 9.3 million hospitalizations and a total cost of BRL $183 billion (US $47.1 billion), ≈BRL $2,000 (US $515.30) per hospitalization, and an average length of stay of 6.9 days (*44*). Infectious diseases accounted for 9% of bed days and 21% of ICU days. In our study, we found ICU expenses comprised 19.6% of chikungunya-related hospitalization costs.

In another study of hospitalization costs (*45*), the authors estimated the cost of 256 chikungunya hospitalizations in Rio de Janeiro in 2019 and found costs totaled BRL $88,926.72 (US $23,235.39), a small share of the BRL $279.8 million (US $73.1 million) in total direct and indirect chikungunya-related costs. In our study, the cost of chikungunya-related hospitalizations is modest compared with the total estimated SUS hospitalization costs described by others (*44*). Indeed, the literature indicates that indirect costs account for the largest part of the total chikungunya-related cost, particularly during the chronic phase, which can be up to 5 times higher than the direct costs (*46*).

Some limitations of our study are inherent to secondary data sources, such as the quality of data entry and the absence of relevant variables for analyzing the topic of interest. The variables of sex and date of birth had no missing or inconsistent values. However, race/skin color was missing in 18% of chikungunya-related hospitalizations; this category was previously documented (*47*) and can distort observed differences between racial groups. Moreover, we could not identify whether the same patient was hospitalized >1 time during the study period or was admitted to private healthcare facilities. In addition, data on underlying conditions were not available, so we were unable to assess their role in the risk of hospitalization or death. We were not able to differentiate whether the hospitalizations for chikungunya occurred during the acute, post-acute, or chronic phase of the disease; thus, we were unable to investigate at which stage of chikungunya the risk of hospitalization is highest. Because we focused on chikungunya as the primary diagnosis at admission, this study might not have captured cases admitted during the postacute or chronic phases, particularly when hospitalizations result from the decompensation of underlying conditions. In addition, SIH/SUS lacks data on cause of death, possibly underestimating death related to complications of chikungunya; thus, our analysis was limited to reporting deaths among patients hospitalized due to chikungunya, which likely underestimates the overall mortality rate associated with the disease. Finally, SIH/SUS publicly available data do not include disaggregated cost components, which prevented assessment of the relative contribution of specific services to overall hospitalization costs.

In conclusion, although the SIH/SUS is an administratively collected database primarily intended for recording hospitalizations funded by the SUS and for reimbursing healthcare facilities for services provided, the system serves as a vital source of information on hospitalizations in Brazil, where most hospitalizations occur in SUS-affiliated facilities (*22*). Although chikungunya-related hospitalization costs for SUS are not substantial, SIH/SUS records provide insights into the profile of the most severe cases of the disease, including ICU admissions and CFRs. Those data are particularly valuable for supporting the planning and organization of healthcare facilities to provide appropriate care according to the severity of each case, which will be especially vital in outbreak and epidemic scenarios.

Appendix Additional information on epidemiology of chikungunya hospitalizations, Brazil, 2014–2024.
