# Supplementary material for: Epidemiology of Chikungunya Hospitalizations, Brazil, 2014–2024
Source: Emerg Infect Dis. 2025 Sep;31(9):1718–28. doi: 10.3201/eid3109.250554 (PMC12407205; doi:10.3201/eid3109.250554)
Supplement: Appendix — Additional information on epidemiology of chikungunya hospitalizations, Brazil, 2014–2024. [file 25-0554-Techapp-s1.pdf]

Article DOI: https://doi.org/10.3201/eid3109.250554

EID cannot ensure accessibility for supplementary materials supplied by authors. Readers who have difficulty accessing supplementary content should contact the authors for assistance.

Epidemiology of Chikungunya Hospitalizations, Brazil, 2014–2024

Appendix

Appendix Table 1. Hospitalizations due to chikungunya (n = 7,421) covered by the unified health system, according to demographic and disease-related data, Brazil, 2014–2024*

Year of hospitalizations	No. of hospitalizations	Sex				Age (years)			Length of hospital stay (days)			Intensive Care Unit (ICU)		Deaths	Case-fatality rate
		M		F											
		No.	%	No.	%	Median	Q1	Q3	Median	Q1	Q3	No.	%	No.	%
2014	27	10	37.0	17	63.0	35	19	44	2	1	3	0	–	0	–
2015	76	34	44.7	42	55.3	27	6	48	3	2	5.5	2	2.6	1	1.3
2016	1,476	620	42.0	856	58.0	35	12	61	3	2	4	10	0.7	27	1.8
2017	1,729	787	45.5	942	54.5	28	9	52	2	2	4	14	0.8	20	1.2
2018	577	266	46.1	311	53.9	32	17	48	2	1	4	6	1	1	0.2
2019	635	285	44.9	350	55.1	33	14	55	3	2	5	11	1.7	4	0.6
2020	211	104	49.3	107	50.7	32	16	55	2	1	4	5	2.4	5	2.4
2021	286	130	45.5	156	54.5	27	9	49	3	2	4	5	1.7	0	–
2022	1,037	441	42.5	596	57.5	41	17	63	3	2	4	14	1.4	9	0.9
2023	762	340	44.6	422	55.4	41	16	66	3	2	4	16	2.1	8	1
2024	605	265	43.8	340	56.2	43	13	65	3	2	6	21	3.5	9	1.5
Total	7,421	3,282	44.2	4,139	55.8	34	12	58	3	2	4	104	1.4	84	1.1

*–, not applicable.

Appendix Table 2. Hospitalizations due to chikungunya (n = 7,421) covered by the unified health system, according to the need for intensive care unit admission, sex and age group, Brazil, 2014–2024

	Overall		Male		Female	
Age group, y	No. hospitalizations	No. ICU admissions (%)	No. hospitalizations	No. ICU admissions (%)	No. hospitalizations	No. ICU admissions (%)
0–4	823	22 (2.67)	438	12 (2.74)	385	10 (2.60)
5–9	667	8 (1.20)	361	5 (1.39)	306	3 (0.98)
10–14	631	6 (0.95)	369	2 (0.54)	262	4 (1.53)
15–19	441	3 (0.68)	199	2 (1.01)	242	1 (0.41)
20–24	410	4 (0.98)	188	1 (0.53)	222	3 (1.35)
25–29	386	6 (1.55)	146	3 (2.05)	240	3 (1.25)
30–34	370	1 (0.27)	166	1 (0.60)	204	0
35–39	429	3 (0.70)	155	0	274	3 (1.09)
40–44	399	3 (0.75)	149	2 (1.34)	250	1 (0.40)
45–49	371	4 (1.08)	122	1 (0.82)	249	3 (1.20)
50–54	389	6 (1.54)	133	4 (3.01)	256	2 (0.78)
55–59	345	4 (1.16)	126	2 (1.59)	219	2 (0.91)
60–64	335	3 (0.90)	124	2 (1.61)	211	1 (0.47)
65–69	322	8 (2.48)	122	5 (4.10)	200	3 (1.50)
70–74	292	8 (2.74)	107	2 (1.87)	185	6 (3.24)
75–79	286	2 (0.70)	122	1 (0.82)	164	1 (0.61)
80–84	256	4 (1.56)	126	2 (1.59)	130	2 (1.54)
85–89	156	5 (3.21)	78	4 (5.13)	78	1 (1.28)
≥90	113	4 (3.54)	51	1 (1.96)	62	3 (4.84)

Appendix Table 3. Hospitalizations due to chikungunya (n = 7,421) covered by the unified health system, according to case-fatality, by sex and age group, Brazil, 2014-2024

	Overall		Male		Female	
Age group, y	No. hospitalizations	No. deaths (% CFR)	No. hospitalizations	No. deaths (% CFR)	No. hospitalizations	No. deaths (% CFR)
0–4	823	5 (0.6)	438	3 (0.7)	385	2 (0.5)
5–9	667	0	361	0	306	0
10–14	631	0	369	0	262	0
15–19	441	0	199	0	242	0
20–24	410	3 (0.7)	188	1 (0.5)	222	2 (0.9)
25–29	386	3 (0.8)	146	1 (0.7)	240	2 (0.8)
30–34	370	1 (0.3)	166	1 (0.6)	204	0
35–39	429	0	155	0	274	0
40–44	399	2 (0.5)	149	2 (1.3)	250	0
45–49	371	1 (0.3)	122	0	249	1 (0.4)
50–54	389	1 (0.3)	133	0	256	1 (0.4)
55–59	345	3 (0.9)	126	2 (1.6)	219	1 (0.5
60–64	335	4 (1.2)	124	2 (1.6)	211	2 (0.9)
65–69	322	6 (1.9)	122	4 (3.3)	200	2 (1.0)
70–74	292	6 (2.1)	107	2 (1.9)	185	4 (2.2)
75–79	286	8 (2.8)	122	5 (4.1)	164	3 (1.8)
80–84	256	12 (4.7)	126	4 (4.0)	130	7 (5.4)
85–89	156	16 (12.3)	78	11 (14.1)	78	5 (6.4)
≥90	113	13 (11.5)	51	7 (13.7)	62	6 (9.7)

Appendix Table 4. Cost of hospitalizations due to chikungunya (n = 7,421) covered by the unified health system, in US dollars (US $), by year of hospitalization, Brazil, 2014–2024*

	Year of hospitalization due to chikungunya											
Features	2014	2015	2016	2017	2018	2019	2020	2021	2022	2023	2024	Overall
No. hospitalizations	27	76	1.476	1.729	577	635	211	286	1.037	762	605	7,421
Exchange rate (to US $) in July 1, 2024	0,451020	0,322197	0,310712	0,303511	0,259783	0,261287	0,183727	0,201577	0,189143	0,205842	0,179036	–
Overall cost (US $)												
Total cost	1.727,32	5.993,01	120.336,60	124.960,10	47.252,28	59.165,68	17.772,88	19.848,68	54.452,12	57.416,51	51.820,96	560,746.14
Mean	63,95	78,84	81,53	72,27	81,88	93,17	84,24	69,40	52,51	75,36	85,69	76,26
Median	78,66	62,64	59,16	57,79	47,38	49,75	34,98	38,38	36,01	39,19	35,52	47,38
IQR	57,37	22,30	12,46	9,71	9,85	12,54	10,29	12,50	9,08	14,82	22,40	–
Hospital services (US $)												
Total cost	1.373,43	4.969,59	100.163,20	103.766,04	39.747,60	50.549,71	15.252,21	16.588,65	45.886,58	48.534,76	44.263,89	471.095,67
Mean	50,88	65,41	67,86	60,00	68,89	79,61	72,28	57,99	44,26	63,69	73,15	64,00
Median	62,74	51,26	48,19	47,07	38,21	40,53	28,50	31,26	29,34	31,93	29,20	38,21
IQR	46,64	20,98	12,43	9,71	9,87	12,54	10,29	12,50	9,08	14,82	20,70	–
Professional services (US $)												
Total cost	353,89	1.023,42	20.173,40	21.194,05	7.504,67	8.615,97	2.520,68	3.260,03	8.565,54	8.881,75	7.557,06	89.650,47
Mean	13,12	13,47	13,67	12,26	13,02	13,56	11,94	11,39	8,27	11,65	12,50	12,26
Median	15,92	11,37	10,97	10,71	9,17	9,22	6,49	7,12	6,68	7,27	6,32	9,17
IQR	10,69	0,00	0,00	0,00	0,00	0,00	0,00	0,00	0,00	0,00	0,00	–
ICU cost (US $)												
Total cost	–	507,51	14.651,29	13.665,16	13.182,51	14.728,56	7.476,07	4.342,45	8.790,85	16.302,69	16.265,42	109.912,51
Mean	–	253,76	1.465,13	976,09	2.197,09	1.338,97	1.495,21	868,49	627,92	1.018,92	774,55	1.101,61
Median	–	253,76	743,72	799,14	1.554,54	844,32	1.583,18	868,49	381,96	617,53	515,62	771,43
IQR	–	417,95	1.264,32	966,23	3.133,94	2.001,33	703,64	578,99	453,94	555,77	644,53	–
% of ICU/total hospitalizations	–	8,5	12,2	10,9	27,9	24,9	42,1	21,9	16,1	28,4	31,4	19,6

*Costs covered by the Brazilian government through Sistema Único de Saúde (SUS) and recorded in the Sistema de Informação database. Data obtained from the Brazil Ministry of Health Datasus (https://datasus.saude.gov.br/transferencia-de-arquivos). IQR, interquartile range (Q3,Q1); –, not applicable.

Appendix Table 5. Cost of hospitalizations due to chikungunya (n = 7,421) covered by the Brazilian unified health system, in Brazi Real (R$), by year of hospitalization, Brazil, 2014–2024*

	Year of hospitalization due to chikungunya											
Features	2014	2015	2016	2017	2018	2019	2020	2021	2022	2023	2024	Overall
Hospitalizations	27	76	1,476	1,729	577	635	211	286	1,037	762	605	7,421
Overall cost (BR $)												
Total cost	3,829.81	18,600.45	387,293.06	411,715.21	181,891.33	226,439.43	96,735.29	98,466.97	287,888.65	278,934.88	289,444.34	3,829.81
Mean	141.8	244.7	262.4	238.1	315.2	356.6	458.5	344.3	277.6	366.1	478.6	141.8
Median	174.4	194.4	190.4	190.4	182.4	190.4	190.4	190.4	190.4	190.4	198.4	174.4
IQR	127.2	69.2	40.1	32	37.9	48	56	62	48	72	125.1	127.2
Hospital services (BR $)												
Total cost	3,045.17	15,424.06	322,366.70	341,885.60	153,003.10	193,464.30	83,015.60	82,294.34	242,602.60	235,786.50	247,234.60	3,045.17
Mean	112.8	203	218.4	197.7	265.2	304.7	393.4	287.7	234	309.4	408.6	112.8
Median	139.1	159.1	155.1	155.1	147.1	155.1	155.1	155.1	155.1	155.1	163.1	139.1
IQR	103.4	65.1	40	32	38	48	56	62	48	72	115.6	103.4

Professional services (BR $)												
Total cost	784.64	3,176.39	64,926.36	69,829.61	28,888.23	32,975.13	13,719.69	16,172.63	45,286.05	43,148.38	42,209.74	784.64
Mean	29.1	41.8	44	40.4	50.1	51.9	65	56.5	43.7	56.6	69.8	29.1
Median	35.3	35.3	35.3	35.3	35.3	35.3	35.3	35.3	35.3	35.3	35.3	35.3
IQR	23.7	0	0	0	0	0	0	0	0	0	0	23.7
ICU cost (BR $)												
Total cost	–	1,575.16	47,153.92	45,023.60	50,744.31	56,369.28	40,691.20	21,542.40	46,477.29	79,200.00	90,850.00	1,575.16
Mean	–	787.6	4,715.40	3,216.00	8,457.40	5,124.50	8,138.20	4,308.50	3,319.80	4,950.00	4,326.20	787.6
Median	–	787.6	2,393.60	2,633.00	5,984.00	3,231.40	8,617.00	4,308.50	2,019.40	3,000.00	2,880.00	787.6
IQR	–	1,297.20	4,069.10	3,183.50	12,063.70	7,659.50	3,829.80	2,872.30	2,400.00	2,700.00	3,600.00	1,297.20
% of ICU/total hospitalizations	–	1,575.16	47,153.92	45,023.60	50,744.31	56,369.28	40,691.20	21,542.40	46,477.29	79,200.00	90,850.00	1,575.16

*Costs covered by the Brazilian government through Sistema Único de Saúde (SUS) and recorded in the Sistema de Informação database. Data obtained from the Brazil Ministry of Health Datasus (https://datasus.saude.gov.br/transferencia-de-arquivos). IQR, interquartile range (Q3,Q1); –, not applicable.

Appendix Table 6. Annual hospitalization costs for chikungunya in Brazil (2014–2024), in BRL, inflation-adjusted BRL (2024 values), and USD.

Year of hospitalization	Cost in BRL	Inflation index*	Cost adjusted for inflation to 2024 values	Cost in US$ (1 BRL = 0.179036 USD)
2014	3,829.81	0.753857	6,716.94	1,202.57
2015	18,600.45	0.610287	29,952.07	5,362.50
2016	387,293.06	0.479486	572,994.81	102,586.70
2017	411,715.21	0.436547	591,448.11	105,890.50
2018	181,891.33	0.376082	250,297.37	44,812.24
2019	226,439.43	0.331326	301,464.69	53,973.03
2020	96,735.29	0.303461	126,090.71	22,574.78
2021	98,466.97	0.202813	118,437.37	21,204.55
2022	287,888.65	0.075596	309,651.77	55,438.81
2023	278,934.88	0.042363	290,751.39	52,054.97
2024	289,444.34	–	289,444.34	51,820.96
Overall	2,281,239.42		2,887,249.57	516,921.61

*Using the BRCPI1986 index.

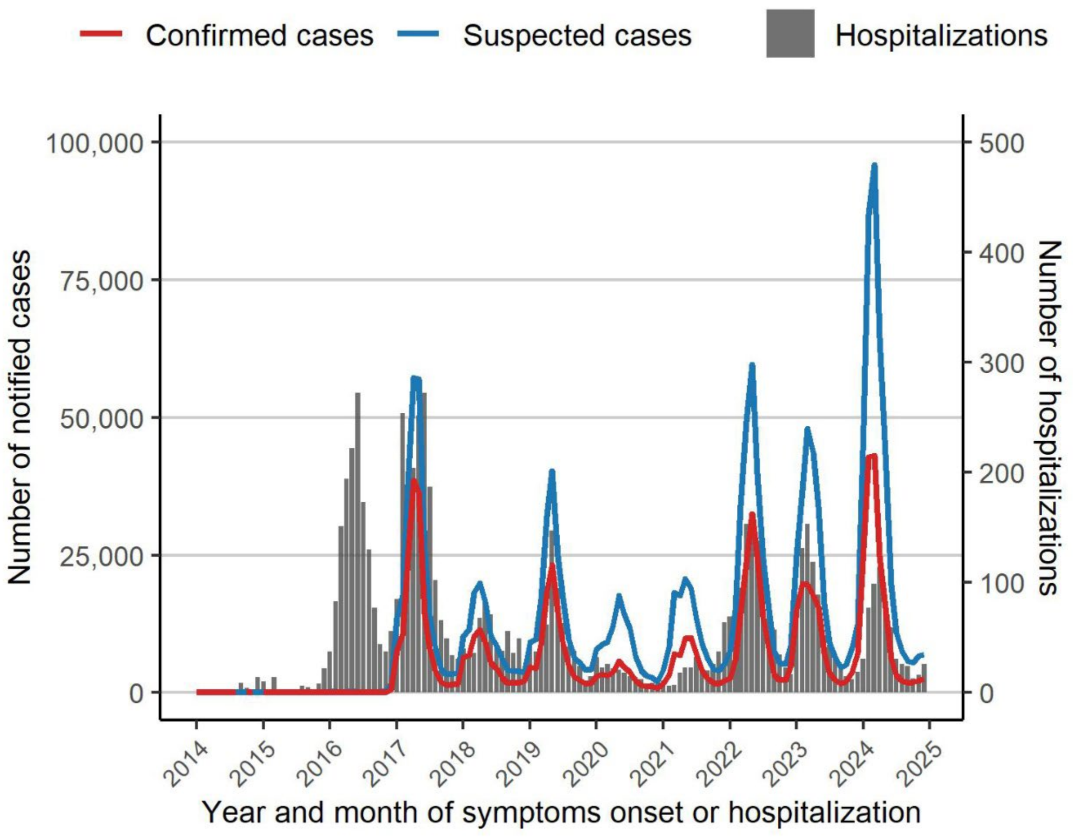

Appendix Figure 1. Time series of reported and confirmed chikungunya cases recorded in the National Disease Notification System (Sinan), by year and month of symptom onset, and of chikungunya hospitalizations recorded in the Hospital Information System of the Unified Health System (SIH/SUS), by year and month of hospitalization, Brazil, 2014–2024.

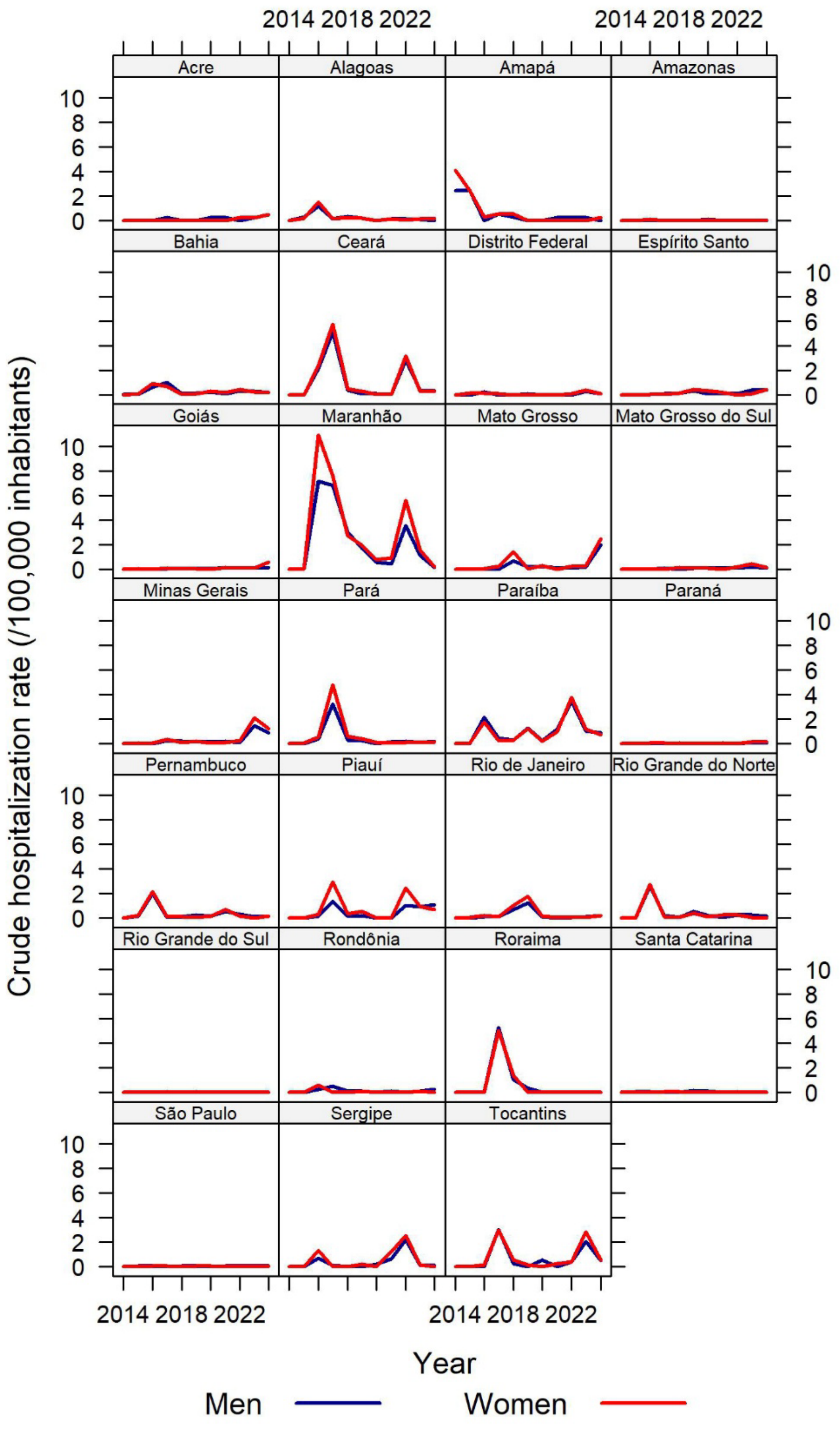

Appendix Figure 2. Crude hospitalization rates due to chikungunya (n = 7,421) covered by the Brazilian unified health system, by year of hospitalization, Federal Unit of residence, and patient sex, Brazil, 2014-2024. Source: Hospital Information System/Unified Health System (SIH/SUS), available in Datasus (https://datasus.saude.gov.br/transferencia-de-arquivos).

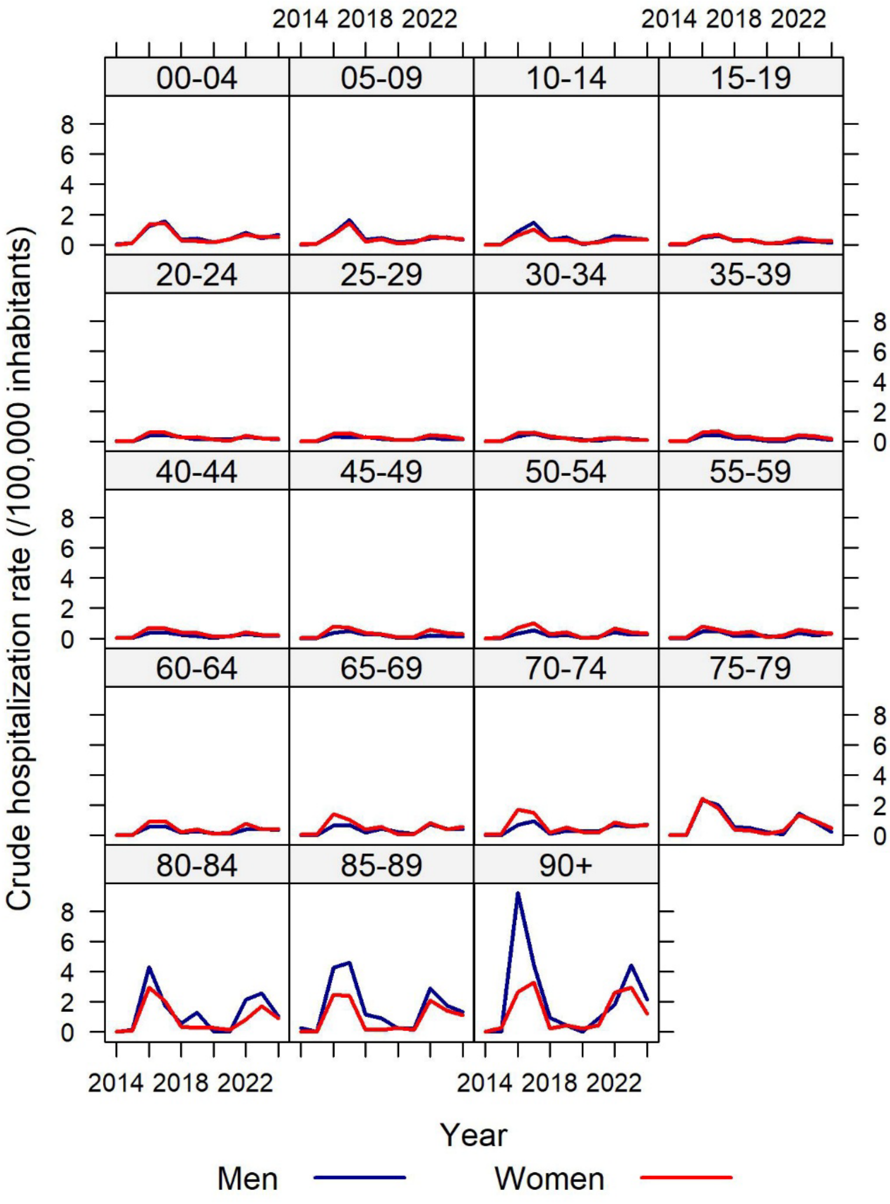

Appendix Figure 3. Crude chikungunya hospitalization rates covered by the Brazilian unified health system (n = 7,421), by age group and patient sex, Brazil, 2014–2024. Source: Hospital Information System/Unified Health System (SIH/SUS), available in Datasus (https://datasus.saude.gov.br/transferencia-de-arquivos).

Age group (Years)	Year of hospitalization 2014	2015	2016	2017	2018	2019	2020	2021	2022	2023	2024	Minigraph
**Overall**												
0-4	0.01	0.11	1.29	1.51	0.29	0.32	0.14	0.35	0.74	0.44	0.57	
5-9	0.01	0.06	0.70	1.54	0.26	0.41	0.12	0.19	0.49	0.47	0.33	
10-14	0.00	0.03	0.77	1.25	0.30	0.41	0.06	0.17	0.48	0.38	0.32	
15-19	0.02	0.03	0.50	0.62	0.26	0.30	0.09	0.13	0.32	0.27	0.18	
20-24	0.02	0.00	0.49	0.51	0.29	0.21	0.14	0.08	0.35	0.22	0.14	
25-29	0.01	0.03	0.42	0.41	0.27	0.23	0.10	0.12	0.35	0.24	0.16	
30-34	0.00	0.03	0.45	0.55	0.28	0.21	0.09	0.12	0.23	0.15	0.10	
35-39	0.04	0.01	0.49	0.55	0.28	0.24	0.09	0.08	0.37	0.28	0.15	
40-44	0.01	0.04	0.53	0.54	0.33	0.25	0.08	0.11	0.35	0.22	0.16	
45-49	0.01	0.03	0.59	0.61	0.31	0.25	0.06	0.06	0.39	0.27	0.18	
50-54	0.00	0.06	0.52	0.78	0.21	0.32	0.03	0.08	0.54	0.32	0.28	
55-59	0.02	0.03	0.63	0.55	0.25	0.33	0.11	0.14	0.47	0.29	0.32	
60-64	0.00	0.01	0.73	0.78	0.19	0.32	0.10	0.12	0.59	0.41	0.39	
65-69	0.02	0.02	1.04	0.86	0.28	0.50	0.11	0.09	0.75	0.39	0.46	
70-74	0.02	0.05	1.25	1.24	0.14	0.43	0.22	0.20	0.76	0.58	0.70	
75-79	0.00	0.03	2.39	1.88	0.44	0.37	0.14	0.19	1.36	0.90	0.37	
80-84	0.00	0.10	3.48	1.93	0.45	0.70	0.17	0.08	1.34	2.07	0.96	
85-89	0.10	0.00	3.14	3.23	0.52	0.42	0.24	0.16	2.39	1.55	1.20	
90+	0.00	0.18	4.90	3.69	0.48	0.46	0.15	0.59	2.34	3.43	1.51	
**Men**												
0-4	0.03	0.12	1.24	1.57	0.32	0.39	0.14	0.35	0.81	0.41	0.65	
5-9	0.00	0.07	0.73	1.63	0.31	0.46	0.16	0.24	0.43	0.50	0.31	
10-14	0.00	0.02	0.90	1.48	0.31	0.48	0.04	0.20	0.59	0.43	0.31	
15-19	0.00	0.05	0.44	0.58	0.28	0.28	0.09	0.13	0.22	0.25	0.12	
20-24	0.04	0.00	0.40	0.44	0.31	0.14	0.14	0.12	0.31	0.22	0.12	
25-29	0.00	0.01	0.33	0.24	0.28	0.18	0.10	0.11	0.26	0.12	0.15	
30-34	0.00	0.05	0.34	0.50	0.24	0.21	0.12	0.06	0.20	0.19	0.10	
35-39	0.03	0.01	0.37	0.41	0.22	0.17	0.06	0.01	0.30	0.23	0.10	
40-44	0.02	0.03	0.37	0.42	0.25	0.14	0.04	0.13	0.29	0.20	0.15	
45-49	0.00	0.03	0.37	0.49	0.25	0.23	0.03	0.02	0.21	0.16	0.10	
50-54	0.00	0.04	0.34	0.52	0.15	0.22	0.03	0.10	0.40	0.23	0.23	
55-59	0.02	0.00	0.44	0.51	0.16	0.19	0.15	0.07	0.37	0.18	0.34	
60-64	0.00	0.03	0.55	0.58	0.17	0.26	0.11	0.07	0.37	0.42	0.33	
65-69	0.00	0.00	0.62	0.67	0.16	0.43	0.21	0.09	0.70	0.43	0.37	
70-74	0.00	0.00	0.70	0.95	0.09	0.30	0.25	0.24	0.66	0.56	0.72	
75-79	0.00	0.00	2.32	1.98	0.55	0.47	0.19	0.06	1.44	0.86	0.22	
80-84	0.00	0.13	4.30	1.76	0.56	1.29	0.00	0.00	2.12	2.56	1.04	
85-89	0.27	0.00	4.25	4.61	1.18	0.91	0.22	0.21	2.90	1.78	1.31	
90+	0.00	0.00	9.22	4.44	0.95	0.46	0.00	0.89	1.80	4.43	2.13	
**Women**												
0-4	0.00	0.10	1.34	1.44	0.27	0.25	0.14	0.36	0.67	0.47	0.49	
5-9	0.01	0.06	0.67	1.43	0.21	0.35	0.08	0.14	0.55	0.44	0.35	
10-14	0.00	0.04	0.64	1.01	0.28	0.34	0.08	0.14	0.37	0.32	0.33	
15-19	0.05	0.01	0.55	0.67	0.25	0.31	0.09	0.14	0.44	0.30	0.25	
20-24	0.01	0.00	0.58	0.58	0.27	0.29	0.14	0.04	0.38	0.23	0.15	
25-29	0.02	0.05	0.52	0.57	0.25	0.27	0.10	0.12	0.45	0.35	0.18	
30-34	0.00	0.02	0.55	0.59	0.33	0.21	0.06	0.17	0.25	0.11	0.10	
35-39	0.05	0.01	0.61	0.67	0.34	0.31	0.13	0.15	0.43	0.33	0.19	
40-44	0.01	0.06	0.69	0.66	0.41	0.36	0.12	0.10	0.41	0.24	0.18	
45-49	0.02	0.03	0.79	0.71	0.37	0.26	0.09	0.10	0.56	0.37	0.26	
50-54	0.00	0.08	0.69	1.01	0.26	0.42	0.03	0.06	0.68	0.40	0.32	
55-59	0.02	0.06	0.80	0.59	0.33	0.44	0.07	0.20	0.57	0.38	0.30	
60-64	0.00	0.00	0.89	0.94	0.21	0.37	0.08	0.17	0.77	0.40	0.44	
65-69	0.03	0.03	1.39	1.02	0.38	0.55	0.03	0.10	0.80	0.36	0.55	
70-74	0.04	0.08	1.70	1.47	0.18	0.53	0.20	0.16	0.84	0.60	0.69	
75-79	0.00	0.06	2.44	1.80	0.36	0.30	0.10	0.28	1.30	0.94	0.49	
80-84	0.00	0.08	2.93	2.04	0.38	0.29	0.28	0.14	0.82	1.73	0.90	
85-89	0.00	0.00	2.50	2.42	0.14	0.13	0.26	0.13	2.08	1.41	1.13	
90+	0.00	0.28	2.66	3.30	0.24	0.46	0.22	0.44	2.59	2.95	1.21	

Appendix Figure 4. Crude hospitalization rates due to chikungunya/100.000 inhabitants covered by the Brazilian unified health system, by year of hospitalization, sex, and age group, Brazil, 2014–2024.
